# Feasibility of point-of-care ultrasound during the emergency department triage: a prospective cohort study

**DOI:** 10.1186/s13089-025-00463-z

**Published:** 2025-11-19

**Authors:** Davide Enrici Baion, Chiara Schettino, Lara Vita, Deborah Luison, Stefania Caprioli, Matteo Lombardo, Alberto La Ferrara, Francesca Locascio, Emanuela Galluzzo, Vittoria Ciampi, Aurora Ianiro, Davide Maserin, Francesco Malara, Noemi Urso, Fulvio Morello, Daniele Marchisio, Enrico Lupia, Emanuele Pivetta

**Affiliations:** 1 Division of Emergency Medicine and High Dependency Unit, Città della Salute e della Scienza di Torino-Molinette Hospital, Corso Bramante 88, Turin, 10126 Italy; 2 School of Nursing, University of Turin, Turin, Italy; 3 Department of Medical Sciences, University of Turin, Turin, Italy; 4 Gruppo Formazione Triage-GFT, Turin, Italy

**Keywords:** Point-of-care ultrasound, Nursing, Emergency department, Triage

## Abstract

**Background:**

The primary aim of this study was to evaluate the feasibility of adding point-of-care ultrasound (POCUS) during the Emergency Department (ED) triage process. This prospective study enrolled two cohorts of adult patients presenting to the ED for a selected group of acute symptoms, previously selected on the basis of the presumed utility of POCUS during triage evaluation. The ED triage process was performed as recommended by the hospital guidelines or by including a nurse-performed POCUS evaluation. Only urgent or less codes were considered eligible for the study. The timing of all evaluations was recorded along with the opinion of the nurses involved in the study on the impact of POCUS results on the triage process. After ED discharge, the most appropriate triage code was determined by independent review of the triage data.

**Results:**

A total of 312 patients were enrolled, 101 of whom were evaluated with the hospital standard triage process. Nine nurses with expertise in both ED triage and POCUS were involved in the study. The majority of the enrolled patients were deferrable or minor urgency (about 60% in both groups). The median time needed for the triage evaluation was 180 seconds (range 540), 90 seconds longer in the POCUS group than in the standard triage group (*p* < 0.01). Net reclassification index of POCUS-implemented compared with standard triage protocol was 8% and 5% for urgent and less urgent cases.

**Conclusions:**

This small single site study suggests that POCUS is feasible during the ED triage and it is potentially useful by triage nurses. However, future studies are needed to confirm POCUS potential usefulness for a more accurate triage process.

## Introduction

Triage in the Emergency Department (ED) is a fundamental process that enables the rapid assessment and categorization of patients based on their clinical priority rather than the order of arrival [1–4]. In the United States of America, approximately 130 million patients are evaluated annually in the EDs, all of whom require a triage assessment [5].

In Italy, ED triage was formally defined in 1992 through a specific national legislation [6, 7], which outlines patient’s classification, the required information to be collected, and the personnel responsible for its execution. Triage is primarily a nursing responsibility and consists of a multi-step process designed to minimize the time between hospital arrival and clinical evaluation, based on the urgency of patient’s clinical conditions.

In the majority of cases, when the so-called “critical look”, i.e. the rapid visual assessment, is not sufficient to determine priority, the triage process required additional evaluation like infection control screening, subjective assessment (i.e., the patient’s self-reported reason for seeking care), guided history-taking by the triage nurse, and objective clinical evaluation [8]. The entire triage process should be completed as quickly as possible. While there is no specific time standard for triage in Italy, international guidelines suggest it should be take no more than 10–15 min [9].

This last step may take advantage of the use of various tools, such as, for instance, electrocardiogram (ECG) [10, 11] or point-of-care ultrasound (POCUS).

POCUS refers to bedside ultrasound evaluation performed by healthcare practitioners to obtain immediate clinical information and to guide patient management [12]. The international literature supports its utility among a broad range of healthcare professionals, including nurses, physician assistants, and physical therapists [12–17]. However, standardized guidelines for its training and application are still lacking [19].

Nursing interest in POCUS originated primarily from the need to rapidly and efficiently place vascular access [19–22]. More recently, nursing applications of POCUS have expanded to include a variety of assessments, such as lung and bladder examination. This reflects the nurse’s evolving role in acute care settings, here including triage, where subtle clinical presentations may mask serious underlying conditions requiring rapid, targeted interventions and POCUS may assist nurses in identifying or excluding significant findings during the objective assessment triage phase.

Therefore, nursing-performed POCUS can be incorporated into the triage process with the aim of identifying conditions at risk of clinical deterioration, rather than establishing a diagnosis. Its applications could potentially include: (1) thorax assessment, to investigate the pleura integrity (e.g., to exclude pneumothorax) or to detect changes in parenchymal density (e.g., congestion or viral pneumonia) or to identify pleural effusion; (2) pelvic assessment, to evaluate bladder volume and urinary retention, as well as to visualize the adjacent structures such as the uterus or the prostate; (3) abdominal assessment, to measure the aortic diameter and to assess the inferior vena cava (IVC) for venous congestion; (4) vascular assessment (via compression technique - CUS), particularly of the lower limbs, to visualize veins and arteries at the inguinal and popliteal level, in order to rule out thrombotic occlusions [17, 22–29].

In this study, we investigated the feasibility of adding POCUS during the triage process and the opinion of the nurses involved in the study on the impact of POCUS results on the triage process. In addition, as secondary aim, we evaluate how the use of POCUS during the ED triage might change the patients’ priority.

## Materials and methods

This was a prospective cohort study conducted at the ED of the Città della Salute e della Scienza di Torino University hospital, Turin, Italy. The study protocol was approved by the institutional review board of the hospital (approval number 507/2022), and written informed consent was obtained from all patients. The study was conducted in accordance with the principles of the Declaration of Helsinki for clinical research involving human subjects.

A group of 12 nurses (seven women and five men) was selected for participating in the study. All of them had (i) a prior training in ED triage, (ii) a minimum of four years of ED work experience, and (iii) more than three years of prior POCUS training. All of them had already completed a 4-hour POCUS course and had performed at least 150 sonographic evaluations before the beginning of the study. These evaluations included bladder assessment, lung ultrasound (assessing sliding, pleural effusion, and parenchymal disease involvement such as presence of B-lines or consolidations), volume status assessments, and ultrasound-guided peripheral intravenous access.

**Fig. 1:**
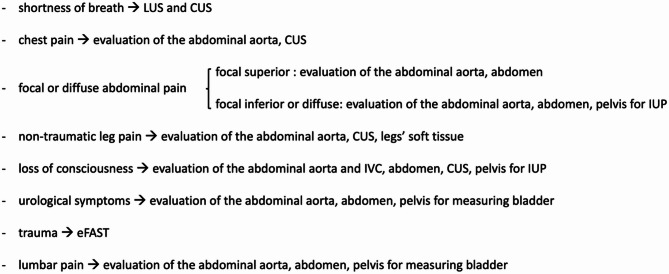
Symptoms included in the study and corresponding point-of-care ultrasound (POCUS) evaluations. POCUS, point-of-care ultrasound; LUS, lung ultrasound (i.e., sliding and lung bases); CUS, compression ultrasonography of leg veins; evaluation of the abdominal aorta included 3 transverse scans and a proximal longitudinal view; abdomen refers to an evaluation similar to the eFAST (extended focused assessment sonography for trauma) scan, for ruling out presence of free fluid; pelvis for IUP (intrauterine pregnancy), evaluation of the pelvis for assessing the presence of a first trimester intrauterine pregnancy; pelvis for measuring bladder refers to a 2-view measurement of the bladder (two transverse diameters, and a longitudinal one). IVC: inferior vena cava. * The LUS evaluation did not include the assessment of changes in parenchymal density (i.e., the presence of B-lines)

Prior to patient enrollment, the participating nurses carried out a consensus-based selection of symptoms to identify patients who might potentially benefit from POCUS during triage. The selected presentations included shortness of breath, chest pain, focal or diffuse abdominal pain, non-traumatic leg pain, loss of consciousness, trauma, lumbar pain, and urological symptoms. Potentially relevant sonographic findings were identified for each symptom category (see Fig. 1).

Before the beginning of the study, each participating nurse also received an additional 4-hour individual training session on basic focused cardiac evaluation (including subcostal view for the FAST exam to detect pericardial effusion in trauma patients), compression ultrasound of lower limb veins (to identify thrombosis or loss of compressibility), abdominal aortic scanning, and identification of intrauterine pregnancy.

During the first period of enrollment, the same nurses enrolled all patients, with and without POCUS examination, but in different days. The control group enrollment period followed that of the POCUS group; however, the nurses were not aware of the triage times associated with the POCUS evaluations.

To evaluate the potential added value of POCUS during the triage process, a second cohort was enrolled after the primary study aim (i.e., feasibility of using POCUS during the triage process) has been assessed. Before the beginning of this second cohort, the study group predefined some specific POCUS findings and their potential impact on the triage code. Patients were always triaged according to the ongoing hospital protocols but an additional code, based on the POCUS findings was also recorded.

After ED discharge, and based on the only clinical data collected during the triage process (i.e., without the POCUS data), two experienced triage nurses (D.E.B. and A.L.F., with 12 and 7 years of triage experience, respectively) independently reviewed each case to determine the “reference” triage code and allocation. In case of disagreement, a third nurse (F.M.) with 16 years of triage experience and national recognition as triage instructor, made the final decision.

**Fig. 2:**
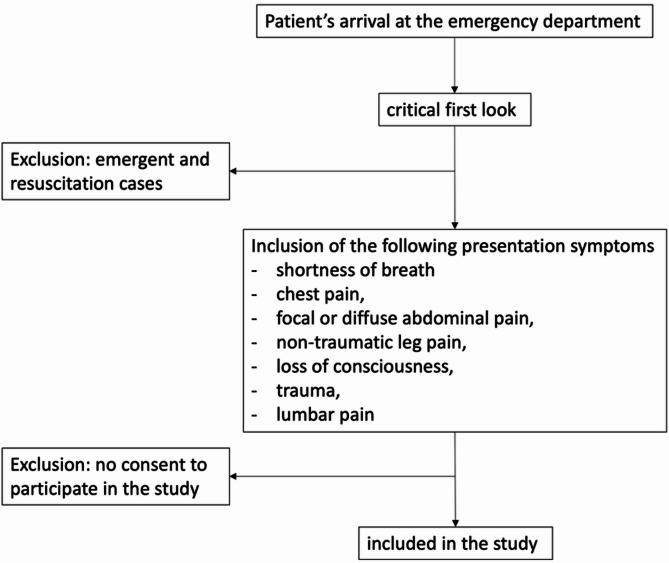
Flow chart of patient enrollment in the POCUS evaluation group *POCUS* point-of-care ultrasound

For the study primary objective, in accordance with the proof-of-concept nature of the study, we planned to enroll a convenience sample of approximately 100 patients evaluated using the standard triage protocol of the Città della Salute e della Scienza di Torino University hospital ED, and another 100 patients evaluated with the addition of POCUS at the time of ED triage (Fig. 2). Patients were excluded if they were classified as emergencies at the initial “critical look” (i.e., assigned as “orange” or “red” code per the Italian triage system, corresponding to emergent or resuscitation status).

The time required to complete the triage evaluation, with or without POCUS (measured from the first clinical question - excluding administrative registration - to the code assignment) was recorded.

POCUS findings were collected using an electronic datasheet.

In the first cohort, after each POCUS evaluation, the triage nurse was also asked to record whether, in his/her opinion, POCUS would have influenced the triage code, using a binary response (i.e., “yes, POCUS could have influenced the triage code” or “no, it could not have influenced the triage code”).

In the second cohort, in addition to the nurse’s subjective judgement, an additional code was recorded according to the new POCUS-integrated protocol previously defined by the researchers’ working group.

For safety reasons, all patients were managed based on the ongoing hospital triage protocols.

As this was a proof-of-concept study, no sample size calculation was performed. Descriptive results are presented as numbers and percentages for categorical variables and mean ± standard deviation (SD) or median with interquartile ranges (25th–75th percentiles) for continuous variables. Comparison between continuous variables were conducted using the Student’s t-test or the Wilcoxon–Mann–Whitney test, and the chi-square test or the Fisher’s exact test for categorical data, as appropriate.

The clinical utility of adding POCUS to the standard triage was evaluated using category-based net reclassification index (NRI) and reclassification tables [31].

POCUS evaluations during triage were performed using Butterfly iQ and iQ + hand-held US devices (Butterfly Network Inc., Burlington, MA, USA) connected to 5th generation iPads mini (Apple Inc., Cupertino, CA, USA).

All statistical analyses were performed using STATA 19 SE (Stata Corp TX, College Station, TX, USA).

## Results

Between March and September 2022, a total of 203 patients were enrolled by 8 nurses. Of these, 101 patients were evaluated using the standard hospital protocol, whereas 102 were assessed with the addition of POCUS during ED triage. In addition, between April and July 2025, 109 additional patients were enrolled and evaluated using the addition of POCUS to the triage process.

The median age of the two cohorts was 57 years (range 18–98) and 56 (18–94), with no statistically significant differences between the two groups (*p* = 0.467) in the first cohort and among them.

The male/female ratio was 0.99 with no differences between the two groups (*p* = 0.833) in the first cohort, and 1.63 in the second cohort.

In terms of ED triage classification, most patients in both groups were categorized as having a deferrable or minor urgency conditions (63 patients and 59, in the standard triage protocol and in the POCUS-implemented group, respectively in the first cohort; 41 in the second cohort). The remaining patients were classified as non-deferrable urgencies (38 and 43 patients, respectively in the first cohort, and 67 in the second cohort—Table 1). A single case was coded as non-urgent in the second cohort.

**Table 1:** Patient characteristics, vital signs, and triage codes reported by study groups and for the both cohorts

	Study groups – first cohort		Total (*N* 203)	*p*-value	Second cohort
	POCUS triage (*N* 102)	Standard triage (*N* 101)			POCUS triage (*N* 109)
Median age (min.-max.)	56.5 (18–93)	59 (18–98)	57 (18–98)	0.467	56 (18–94)
Male/female ratio	0.96	1.02	0.99	0.833	1.63
Arrival to the ED					
Emergency Medicine Service	13.7%	17.8%	15.8%	0.423	18.3%
Self-presenting	86.3%	81.2%	84.2%		81.7%
Past medical history					
Diabetes	11.1%	10.4%	10.8%	0.876	13.2%
Hypertension	30.3%	31.2%	30.8%	0.886	32.1%
Coronary artery disease	12.1%	8.3%	10.3%	0.383	17%
Arrhythmia (any type)	11.1%	12.5%	11.8%	0.764	11.3%
Cerebrovascular disease	8.1%	6.3%	7.2%	0.621	2.8%
Dyslipidemia	14.1%	18.8%	16.4%	0.385	16%
Neoplasia (past or active)	18.2%	22.9%	20.5%	0.413	14.3%
COPD or asthma	12.1%	7.3%	9.7%	0.256	12.7%
Chronic renal failure	7.1%	3.1%	5.1%	0.331*	5.7%
Previous PE or DVT	4%	8.3%	6.2%	0.246	2.8%
Potential COVID-19 related symptoms	6.1%	3.1%	4.6%	0.498	0.9%
Vital signs					
Systolic blood pressure, mmHg, median (min-max)	140 (100–205)	130 (98–200)	135 (98–205)	0.225	135 (100–180)
Diastolic blood pressure, mmHg, median (min-max)	80 (50–130)	75 (55–110)	80 (50–130)	0.183	80 (45–110)
Heart rate, bpm, median (min-max)	83 (45–140)	75 (55–150)	80 (45–150)	0.06	80 (55–130)
Body temperature, °C, median (min-max)	36 (35-38.6)	36 (35.7–29)	36 (35–39)	0.165	36.1 (35–39)
Oxygen saturation in room air, %, median, (min, max)	97.5 (96–100)	97.5 (95–100)	97.5 (95–100)	0.781	98 (93–100)
ED disposition					
Discharge	76.6%	85%	80.9%	0.136	88.6%
Admission	23.4%	15%	19.1%		11.4%
Triage code (first assignment)					
Urgent	37.6%	42.2%	39.9%	0.470	37.6%
Less urgent	63.4%	56.8%	59.6%		61.5%
Non urgent	-	1%	0.5%		0.9%
ED allocation					
Medicine	52.5%	58.8%	55.7%	0.363	78.9%
Surgery	47.5%	41.2%	44.3%		19.3%
Outpatient clinic	–	–	–		1.8%

*POCUS* point-of-care ultrasound,* ED* emergency department,* COPD* chronic obstructive pulmonary disease,* PE* pulmonary embolism,* DVT* deep venous thrombosis,* COVID-19* Coronavirus disease 2019,* °C* Celsius degree,* min.* minimum,* max.* maximum

In both groups of the first cohort, the most frequently reported symptom was abdominal pain (38 patients in the standard triage group and 33 in the POCUS-implemented group). In the standard triage group, this was followed by non-traumatic leg pain and chest pain (17 and 16 patients, respectively). In the POCUS-implemented group, shortness of breath and non-traumatic leg pain were the next most common symptoms (20 and 16 patients, respectively). In the second cohort, the most common symptom was chest pain (32 patients), followed by shortness of breath and non-traumatic leg pain (25 and 22 patients, respectively). Detailed symptom distributions in the two cohorts are presented in Table 2.

**Table 2:** Main symptoms reported at emergency department presentation by study groups and for both cohorts

Symptoms at presentation, *N* (%)	Study groups – first cohort		Total (*N* 203)	Second cohort
	POCUS triage (*N* 102)	Standard triage (*N* 101)		POCUS triage (*N* 109)
Shortness of breath	21 (20.6)	5 (4.9)	26 (12.8)	25 (22.9)
Urological symptoms	10 (9.8)	13 (12.7)	23 (11.3)	5 (4.6)
Abdominal pain (focal and diffuse)	33 (32.4)	38 (38.2)	71 (35.3)	15 (13.8)
Non-traumatic leg pain	16 (15.7)	17 (16.7)	33 (16.2)	22 (20.2)
Lumbar pain	3 (2.9)	7 (6.9)	10 (4.9)	6 (5.5)
Chest pain	12 (11.8)	16 (15.7)	28 (13.7)	32 (29.4)
Loss of consciousness	3 (2.9)	3 (2.9)	6 (2.9)	2 (1.8)
Trauma	4 (3.9)	2 (2)	6 (2.9)	2 (1.8)

*POCUS* point-of-care ultrasound

The median time required to complete the triage evaluation (measured from the first nurse interaction following the administrative registration) was 150 s in the standard triage group and 240 s in the POCUS-implemented group (*p* < 0.01). In the second cohort, the median duration was again 240 s. Table 3 shows the time required for the evaluation across specific presenting symptoms in all groups. As shown, there was a considerable variability in the evaluation time, with differences between the two groups ranging from 60 to 200 s in both cohorts.

**Table 3:** Median time required for triage assessment by presenting symptom (post-administrative registration) in each study group and for the entire cohort

Median time needed for symptom group at presentation, seconds (min, max)	Study groups – first cohort		Total (*N* 203)	*p*-value	Second cohort
	POCUS triage (*N* 102)	Standard triage (*N* 101)			POCUS triage (*N* 109)
Shortness of breath	240 (120–600)	120 (100–180)	240 (100–600)	0.02	180 (120–500)
Urological symptoms	240 (120–360)	120 (60–240)	180 (60–360)	0.004	240 (180–360)
Abdominal pain (focal and diffuse)	240 (120–480)	120 (60–300)	180 (60–480)	< 0.001	180 (120–300)
Non-traumatic leg pain	300 (120–420)	120 (60–220)	180 (60–420)	< 0.001	180 (120–300)
Lumbar pain	240 (120–350)	180 (60–240)	180 (60–350)	0.296	210 (120–300)
Chest pain	240 (120–520)	180 (120–480)	180 (120–520)	0.02	240 (120–600)
Loss of consciousness	380 (180–500)	180 (180–240)	210 (180–500)	0.246	375 (300–450)
Trauma	202.5 (120–400)	140 (100–180)	180 (100–400)	0.240	180 (120–240)
Overall	240 (120–600)	146.5 (60–420)	180 (60–600)	< 0.01	240 (120–600)

*POCUS* point-of-care ultrasound,* min.* minimum,* max.* maximum

Most patients in all groups were discharged from the ED, 79 in the standard triage group and 87 in the POCUS-implemented group, respectively (*p* = 0.208 between the two groups of the first cohort), and 93 in the second cohort.

In the first cohort, in 31 cases (30.4% of patients evaluated with POCUS), the nurses reported that POCUS findings would have led to a different triage code compared to that assigned based solely on clinical assessment. In the second cohort, this opinion was expressed in 22 cases (20.2%).

The two reviewers agreed on the assigned code in 65 cases (59.6%). Among these, they assigned 2 emergent (3.1%), 19 urgent (29.2%), 41 less urgent (63.1%), and 3 non-urgent (4.6%) cases. For the remaining cases, the third reviewer provided the final classification: 1 emergent (2.3%), 17 urgent (38.6%), 25 less urgent (56.8%), and 1 non-urgent (2.3%) case.

The NRI of POCUS-implemented approach compared to the standard triage process for emergent case was 33.3%, for the urgent cases 8%, for less urgent cases 5%, and for the non-urgent cases 25%.

Specifically, the POCUS-implemented approach upgraded the assigned triage code from less urgent to urgent in 9 cases (1 absence of lung sliding, 3 bilateral pleural effusion, 2 acute urinary retentions, 1 abdominal aortic aneurysm without effusion, and 2 cases of lower-limb soft tissue edema). Conversely, it was downgraded from urgent to less urgent in 7 cases (all showing no pathological POCUS findings, including negative eFAST examinations). On the other hand, POCUS-implemented approach upgraded the code from less urgent to urgent in 4 cases (2 positive CUS findings, and 2 unilateral pleural effusions), and downgraded it from urgent to less urgent in 6 cases (no pathological POCUS findings – see Table 4).

**Table 4:** Net reclassification index (NRI) for each triage code as defined after the revision process in the Secondo cohort

Emergent		Standard triage		Total
		Emergent	Urgent	
POCUS triage	Emergent	0	1	1
	Urgent	0	2	2
	Total	0	3	3

Urgent		Standard triage		Total
		Urgent	Less urgent	
POCUS triage	Urgent	16	9	25
	Less urgent	6	5	11
	Total	22	14	36

Less urgent		Standard triage		Total
		Urgent	Less urgent	
POCUS triage	Urgent	7	4	13
	Less urgent	7	48	53
	Total	14	52	66

Non urgent		Standard triage		Total
		Less urgent	Non urgent	
POCUS triage	Less urgent	2	0	2
	Non urgent	1	1	2
	Total	3	1	4

Regarding patient allocation, reviewers agreed in 94 cases, while the third reviewer adjudicated the remaining 15. The POCUS approach reassigned 5 of the 6 cases that were misallocated by the standard process (the remaining case was assigned to the medicine ED instead of the outpatient clinic by both approaches, as defined by the reviewers).

## Discussion

The findings of the present study suggest that incorporating POCUS into the standard ED triage process is feasible. Moreover, our results show that the addition of POCUS during triage increases the evaluation time compared to the standard ED triage protocol. However, according to the nurses involved in the study and the data of the second cohort, POCUS findings could lead to assigning a different triage code, suggesting that this additional time might be associated with a more accurate triage process, including a more accurate “care pathway”.

Nurse-led triage is the standard approach for the initial assessment of patients presenting to the ED [6]. Triage is a complex task that involves multiple, almost simultaneous, steps, some of which are inherently subjective [32, 33]. Additionally, many ED presentations involve symptoms classified as deferrable or non-urgent, making it challenging to promptly identify those requiring immediate care despite seemingly benign complaints [34, 35].

To our knowledge, this is the first study evaluating the feasibility of integrating POCUS into the ED triage process and its potential clinical utility.

Unsurprisingly, the use of POCUS led to an increase in triage duration, even if in our protocol, POCUS assessments were guided by patient’s specific symptoms (e.g., in a patient presenting with shortness of breath, we performed lung ultrasound to evaluate pleural sliding and effusion, along with compressive ultrasound of the leg veins to rule out deep vein thrombosis - see Fig. 1). In addition, we tried to limit the POCUS examinations to those promptly useful for accurately defining each patient’s code. For example, we did not include B-lines detection finding in the POCUS triage approach, as we considered it of limited usefulness not in general but in the triage setting and believed that its evaluation would significantly prolong the triage process. Our intention was not to suggest that the detection of B-lines is without clinical value, but rather that it would contribute to diagnosis—an aim beyond the scope of triage. Moreover, any protocol for evaluating lung parenchyma would be too time-consuming to be feasible during triage.

In our study, we also recorded the opinion of the nurses involved in the study on the impact of POCUS results on the triage process. In approximately one-third quarter of cases, triage nurses reported that POCUS findings could have modified the triage code initially assigned based solely on clinical evaluation. Since participating nurses had extensive triage experience, it is conceivable that the impact of POCUS might be even greater among less experienced operators. Therefore, in the second cohort of the study, we tested the hypothesis that the observed moderate increase in triage time could be outweighed by the potential clinical benefits of adding POCUS to the ED triage process.

However, such an objective inherently faces a conceptual issue concerning the definition of the “appropriate” triage code. According to Italian triage regulations, the correct code should ensure that each patient receives the appropriate level of care at the right time and in the right setting.

This seems to be a reasonable definition but a universally accepted definition of “appropriate” in this context is lacking. This implies that the reference standard for triage coding is, in fact, far from being a true “gold standard.” For this reason, we chose a “surrogate” of it as reference test, the agreement between two expert triage nurses after the ED discharge.

In all enrolled cases, the total waiting time between triage and clinical assessment ranged between 60 and 240 min. The addition of 1.5 min (*p* < 0.01) required for POCUS during triage is unlikely to delay patient care or negatively impact outcomes. However, the potential for a “cascade effect”, where increased triage time results in a backlog for subsequent patients, must be also taken in consideration. A formal risk/benefit analysis is necessary to determine whether the potential improved triage accuracy justifies the delay in triage completion.

More generally, based on the model we adopted (the so called “comprehensive triage model”), the benefit of using POCUS would be considered not only in terms of changes in triage code but also in relation to a more appropriate overall care pathway.

Since POCUS is an operator-dependent tool and its effective use requires dedicated training, which, in our study, was even reinforced before the study began, this aspect may represent an important limitation for the generalizability of our results, especially during the early phases of POCUS implementation in ED triage.

Despite the promising nature of our findings, several limitations should be acknowledged. First, our study was conducted at a single center and it is a proof-of-concept study. Both these aspects may limit the generalizability to other institutions and settings. Further multicenter studies are needed to validate these results across different settings, including pediatric populations. Second, the limited sample size precludes definitive conclusions about the utility of adding POCUS in ED triage.

This was estimated using the NRI based on the already metioned “imperfect” reference standard. This helps explain some of the apparent errors made by the POCUS-implemented approach in modifying the triage code.

For instance, in two cases POCUS identified the presence of deep vein thrombosis (DVT), which would have warranted an upgraded triage code. However, since no other triage assessment method could detect DVT during the process, the reviewers, without this information and following the hospital standard protocols in use, considered these code increases as misclassifications by the POCUS-implemented approach.

A similar situation occurred when the triage code was downgraded from urgent to less urgent in the absence of pathological POCUS findings (e.g., with an eFAST evaluation negative for free fluids and for pnemothorax).

A third potential limitation is related to our exclusion criteria. Emergent cases were excluded from the study, as they are typically classified and managed immediately upon arrival. This limits our findings to patients with less critical presentation.

Fourth, a potential limitation may be related to the hand-held machines we used. However, previous studies have shown a good overall agreement between hand-held and high-end ultrasound machines [36, 37].

Finally, the POCUS expertise of participating nurses exceeded what is commonly seen in clinical practice. This could have influenced both the efficiency with which they completed the triage process and their perceived usefulness of POCUS.

In conclusion, our small, single site study provides preliminary evidence supporting the feasibility of incorporating POCUS into the ED triage process. While POCUS use extends evaluation time, POCUS might enhance triage accuracy and facilitate early recognition of specific conditions at risk of rapidly deteriorating. Further research is needed to confirm these findings, in particular to clarify the potential POCUS utility in the ED triage, based on a new and universally recognized “gold standard” for the triage evaluation, and to guide the development of standardized protocols for its safe and effective implementation in ED triage.

## Data Availability

The datasets used and/or analyzed during the current study are available from the corresponding author on reasonable request.

## Abbreviations

CUS: Compression ultrasonography
ED: Emergency department
ECG: Electrocardiogram
FAST: Focused assessment sonography for trauma
IVC: Inferior vena cava
NRI: Net reclassification index
POCUS: Point-of-care ultrasound
SD: Standard deviation
